# Vulnerability situations of adolescents and young people from rural areas undergoing cancer treatment*

**DOI:** 10.1590/1980-220X-REEUSP-2024-0250en

**Published:** 2025-02-10

**Authors:** Fernanda Duarte Siqueira, Graciela Dutra Sehnem, Nara Marilene Oliveira Girardon-Perlini, Aline Cammarano Ribeiro, Cíntia Beatriz Goi, Camila Lovato de Figueiredo, Eliane Tatsch Neves

**Affiliations:** 1 Universidade Federal de Santa Maria, Departamento de Enfermagem, Santa Maria, RS, Brazil.; 2 Universidade Federal de Santa Maria, Programa de Pós-Graduação em Enfermagem, Santa Maria, RS, Brazil.

**Keywords:** Adolescent, Medical Oncology, Rural Population, Health Vulnerability, Adolescente, Oncología Médica, Población Rural, Vulnerabilidad en Salud

## Abstract

**Objective:**

To understand the vulnerability situations of adolescents and young people from rural areas undergoing cancer treatment

**Method:**

Qualitative research, mediated by the health vulnerability framework, developed in an oncology service in southern Brazil with adolescents and young people from rural areas undergoing cancer treatment, through semi-structured, audio-recorded interviews, from April 2021 to September 2022, taking place in five phases, with data analysis carried out in light of Reflexive Inductive Thematic Analysis.

**Results:**

Eleven adolescents and four young people participated in the study, the majority of whom were male, single, with a family income of up to three minimum wages and a diagnosis of Acute Lymphoblastic Leukemia. The vulnerable situations of adolescents and young people in rural areas undergoing cancer treatment involve challenges such as the emotional impact of the diagnosis, loss of contact with friends, side effects of chemotherapy, dietary restrictions, access, and travel to undergo treatment.

**Conclusion:**

Adolescents and young people undergoing cancer treatment face situations of vulnerability related to the disease, limited access to adequate health care, and social and emotional difficulties.

## RESUMEN

**Objetivo:**

Comprender las situaciones de vulnerabilidad de adolescentes y jóvenes de contextos rurales en tratamiento contra el cáncer.

**Método:**

Investigación cualitativa, mediada por el marco de vulnerabilidad en salud, desarrollada en un servicio de oncología del sur de Brasil con adolescentes y jóvenes de zonas rurales en tratamiento contra el cáncer, a través de entrevistas semiestructuradas audiograbadas, de abril de 2021 a septiembre de 2022, que se desarrolló en cinco fases, con análisis de datos realizado a la luz del Análisis Temático Inductivo Reflexivo.

**Resultados:**

Participaron del estudio 11 adolescentes y cuatro jóvenes, en su mayoría hombres, solteros, con ingresos familiares de hasta tres salarios mínimos y diagnosticados con Leucemia Linfoblástica Aguda. Las situaciones de vulnerabilidad de adolescentes y jóvenes de contextos rurales en tratamiento contra el cáncer implican desafíos, como el impacto emocional del diagnóstico, la pérdida de contacto con amigos, los efectos secundarios de la quimioterapia, las restricciones dietéticas, el acceso y los viajes para someterse al tratamiento.

**Conclusión:**

Los adolescentes y jóvenes sometidos a tratamiento contra el cáncer enfrentan situaciones de vulnerabilidad relacionadas con la enfermedad, acceso limitado a una atención médica adecuada y dificultades sociales y emocionales.

## INTRODUCTION

Adolescence and youth correspond to biological processes, cultural, political-economic, and territorial constructions that throughout different eras and historical processes have acquired different meanings and conceptions^(1)^. The World Health Organization (WHO) refers to the terms adolescents and young people whose age range includes people from 10 to 24 years old^(2)^, a definition used in the construction of this study. In these phases, oncological treatment can interfere in the process of formation, development, and maturation, producing repercussions on daily life and leisure activities^(3)^.

In Brazil, cancer is now the main cause of death from disease among people aged 19 or younger. Approximately 6% of all cancers affect adolescents and young people between the ages of 15 and 29. Considering this scenario, 80% of children, adolescents, and young people can be cured if diagnosed early and if they receive appropriate treatment in specialized centers^(4)^.

In this scenario, the reality of the individual undergoing cancer treatment can be influenced by the sociocultural, economic, and life context of those involved, presenting particularities that may be related to the fact that they live in urban or rural areas^(5)^. In this regard, the rural environment can be understood as a way of life that encompasses all members who live and work there^(6)^. Living in this environment presents specific challenges, such as the lack of adequate infrastructure, roads and public transport, making access to health services difficult, impacting the lives of adolescents and young people, and potentially worsening their health conditions during cancer treatment^(7)^. A study investigating rural-urban disparities showed that in rural areas early detection and access to cancer treatment is a challenge, resulting in worse health outcomes^(4)^.

The growth and development of Brazilian adolescents and young people are often permeated by a context of vulnerability such as poverty, sexually transmitted infections, chronic diseases, among others^(8)^. In this context, vulnerability is summarized as an expanded and reflective perception that considers the chance of people being exposed to illness and the consequent impacts on dynamic wholes, encompassing a set of aspects that are not only individual, but also collective, contextual, cultural, economic, and political that lead to susceptibility to illness^(9)^.

To this end, vulnerability involves the articulation of three components: the individual, the social, and the programmatic ones. The individual refers to the degree and quality of information that individuals have about the problem and the possibility of using it for protection. The social component encompasses aspects that depend on access to the media, availability of material resources, and political factors. The programmatic component is related to the actions of programs aimed at prevention and care, and may encompass local, regional, and national policies that must be made effectively and democratically available^(9)^.

Nursing is part of this context as a profession with an important role in the development of care actions for adolescents and young people, given its role in the prevention and promotion of health, in cancer care and treatment^(10)^. Thus, health professionals, especially nurses, are on the front line of health services and need to consider situations of vulnerability and the characteristics of the scenarios in which these people are inserted^(11)^.

A review study that aimed to identify and characterize trends in Brazilian scientific production regarding the health of adolescents and young people living in rural settings highlighted the existing gap in knowledge in this area. The evidence revealed that the topic is still in its infancy, with few studies in which the main topics addressed were related to aspects of health risk, harmful habits and lifestyles, such as inadequate nutrition, associated with chronic non-communicable diseases. In addition, health determinants, physical activity, health promotion needs, access to and use of services, as well as cultural, gender, sexuality, reproduction and violence perspectives were addressed^(11)^, which justifies the importance of deepening research into these topics to better understand and meet the needs of this audience. Based on this, the question was: what are the situations of vulnerability that adolescents and young people in rural areas undergoing cancer treatment experience? Therefore, the objective of this study was to understande the vulnerability situations of adolescents and young people from rural areas undergoing cancer treatment

## METHOD

### Design of Study

Qualitative, descriptive, and exploratory study^(12)^ that meets the recommendations of the *Consolidated criteria for reporting qualitative research* (COREQ), Portuguese version^(13)^, mediated by the health vulnerability framework^(9)^.

### Local

The study was carried out in an oncology department of a general public teaching hospital in southern Brazil.

### Population and Selection Criteria

Adolescents aged between 10 and 19 years and young people aged up to 24 years were selected for the study, as defined by the WHO^(14)^, who were undergoing cancer treatment or clinical follow-up for a maximum of five years (after this period, the person can be considered cured and a cancer survivor)^(15)^, regardless of the type of cancer, who lived in a rural area at the time of diagnosis. Among the population, those who did not have clinical conditions to participate in the study according to the indication of the oncology service team were excluded.

### Data Collection

Data collection took place in person from April 2021 to September 2022. The study was developed in five phases. In the first phase, the professionals of the services were approached, the research proposal was presented to the team along with the importance of developing it, there was the recognition of and familiarization with the routine of the field of study, monitoring of the oncological treatment, and establishment of a bond. The routine of adolescents and young people undergoing cancer treatment varied depending on the type of cancer, stage of the disease, and the specific treatment plan.

In the second phase, participants were recruited, using the list of hospitalized patients and the weekly care schedule. In addition, data were collected from the medical records of adolescents and young people, via the Management Application for University Hospitals (AGHU), accessing scheduled consultations to select participants and collect sociodemographic data such as: sex, age, marital status, origin, address, education, whether they are going to school, whether they are working, family income, medical diagnosis, length of treatment, date of start of treatment, proposed treatment, treatment status, relapse, and displacement. To this end, intentional sampling was used, recruiting all adolescents and young people who lived in rural areas.

Subsequently, in the third phase, professionals from oncology services were asked to help establish the first contact between the adolescents, young people and the researcher, informing them about the study and/or indicating possible participants. In the fourth phase, after identifying the participants who met the selection criteria, personal contact was established between the researcher, the adolescent and/or young person and their family member or legal guardian during hospitalization, or on the day of the scheduled chemotherapy treatment or medical consultation. Accordingly, an invitation was made to participate in the study. In the fifth phase, the research continued through face-to-face interviews, with a semi-structured script, guided by the following guiding questions: “What is it like for you to undergo treatment?”; “How did you discover your illness?”; “Who has helped you during treatment?”; “How do you get around your treatment routine?”; “How does treatment affect your leisure time, school, work, friends and family?”. The interviews were conducted only with the presence of the researcher and the participant, at which time the genogram and ecomap were constructed, used as tools to approach the participants. Interviews were stopped when data were saturated.

The interviews were audio-recorded, took place before and during the chemotherapy infusion or after the medical consultation, all in the morning, and had an average duration of 52 minutes in a room or reserved space available and preferred by adolescents and young people. There were no withdrawals after acceptance to participate in the interview. As data collection took place during the COVID-19 pandemic, biosafety and contamination prevention measures were followed by the researcher and interviewees.

### Data Analysis And Treatment

The interviews were transcribed, organized, and subjected to the Inductive Reflexive Thematic analysis proposed by Braun and Clarke (2019)^(16)^, consisting of six phases: 1) Familiarizing with data; 2) Generating initial codes; 3) Searching for themes, grouping codes into potential themes, and gathering all relevant data for each potential theme; 4) Reviewing identified themes; 5) Defining and naming themes; 6) Producing the report. In this way, data were produced and grouped around a thematic category and four subthemes highlighted and presented in the results.

As the objective of the present study was to understand the phenomenon of oncological treatment, despite the difference in age between younger participants and those considered “older”, situations of vulnerability could be identified, considering the homogeneity and heterogeneity among both adolescents and young people in the rural setting.

### Ethical Aspects

The study followed all the guidelines and legal prerogatives established by Resolutions No. 466/2012 and 510/2016 of the National Health Council. It was approved by the Research Ethics Committee (CEP) with registration number 4.594.079. All participants were informed in clear and objective language about the nature of the research, its objectives, and how to proceed to respond to the interviews. The Free and Informed Consent Form (FICF) was provided to adolescents and young people over 18 years of age and the Assent Form to participants under 18 years of age, subject to the signature of their parents or legal guardian, in two copies, one for the participant and the other for the researcher. The anonymity of study participants was preserved through the adoption of codes, replacing their names with the letter P, in the order in which the interview was conducted (P1, P2 ... P15).

## RESULTS

Eleven (73.3%) adolescents and four young people (26.7%) participated in the study, with an average age of 15.6 years. Among adolescents, ages ranged from 10 to 19 years and among young people, from 20 to 22 years. Among the adolescents, seven (63.7%) were male and four (36.3%) were female. Among the young audience, three (75%) were male and one (25%) participant was female. Thus, it can be summarized that 10 (66.7%) participants were male and five (33.3%) were female. The participants came from 15 rural locations in 13 municipalities in the State of Rio Grande do Sul, predominantly from the central-west region of the State.

Regarding marital status, 10 (66.7%) declared themselves to be single, three (20%) to be dating, and two (13.3%) to be married. Regarding education, five (33.3%) had finished High School, five (33.3%) had unfinished primary education, three (20%) had unfinished high school, and two (13.3%) had finished primary education. Regarding school activities, among adolescents, six (54.5%) interrupted their studies during treatment, three (27.3%) were participating remotely, and two (18.2%) attended school. Of the young participants, two (50%) dropped out of college, from the courses of administration and animal science. Among adolescents and young people, seven (46.7%) were employed and all stopped working due to treatment.

In this context, it can be seen that the family income was up to three minimum wages. Regarding medical diagnosis, it was found that nine (60%) of the participants were diagnosed with Acute Lymphoblastic Leukemia, four (26.7%) with Acute Myeloid Leukemia, one with Hodgkin’s Lymphoma (6.7%), and one (6.7%) with Non-Hodgkin’s Lymphoma. As for treatment, the use of chemotherapy predominated. Regarding the treatment status, eight (53.3%) were undergoing clinical monitoring, two (13.3%) in the induction phase, two (13.3%) in the maintenance phase, one (6.7%) in the consolidation phase, one (6.7%) undergoing treatment for transplantation, and one (6.7%) undergoing diagnostic evaluation due to relapse. It should be noted that relapse occurred in three participants. Treatment time ranged from three to 60 months.

The majority (46.7%) of participants traveled to undergo oncological treatment using the transport provided by the Municipal Health Department of their reference municipality, five (33.3%) traveled using their own means (car or bus), and three (20%) participants alternated between transport provided by the Municipal Health Department and their own means.

From the analysis of the results, situations of vulnerability experienced by the participants were identified based on challenges of cancer treatment, constituting the following thematic category: “Challenges of cancer treatment for adolescents and young people in rural areas”.

Adolescents and young people in rural areas experience cancer treatment, facing several challenges from the moment they discover they have cancer to the various obstacles involved, which predispose them to individual, social, and programmatic vulnerability. The occurrence of disruption in daily life, a new unknown, restrictive and lasting reality, imbalances in emotional bonds, negative impacts due to the side effects of cancer treatment, and difficulties in access were revealed.

*Shock due to the unknown: “I didn’t know leukemia was a cancer”*

The narratives of the adolescents and young people in this study reveal that the discovery of cancer is permeated by lack of knowledge or stigmatized information, associated with the social representation of the disease, producing feelings of fear, concerns even of death, signaling an individual vulnerability, as reported in the following statements:

(...) *they didn’t tell me I had cancer, they said it was leukemia. I didn’t know leukemia was a cancer. The doctor said, “You have very severe anemia and it could turn into leukemia.” Then my sister who was with me started crying and I didn’t understand anything, and I said “why are you crying, I’m just going to take some saline solution and leave”*. (P3)

(...) *I didn’t know what it was* (...) *I didn’t really know what was happening, I’d never heard of it, at first I thought it was something really bad and it was really bad* (...) *it was a death situation. I couldn’t brush my teeth because blood came out, I thought I was going to die.* (P10)

(...) *it was a “boom” when the doctor told my father what I had. I knew leukemia was not good, but I didn’t imagine it was cancer. I was scared, not knowing what to expect. And for me, leukemia was a little thing in the blood, I didn’t pay much attention to it. It seems that the moment you find out, everything goes wrong, it goes from bad to worse.* (P11)

These situations, arising from the statements, occur because they do not recognize, or have not been informed, or clarified about their current condition, or because they do not understand the magnitude of their illness, or do not understand how the treatment could occur and do not expect to experience this process of illness during adolescence and youth.

(...) *the moment he* [the doctor] *said that the treatment is not short but long, that’s when it became important.* (P5)

*It was a shock, actually, I was taking it as if it were the flu, a two, three month treatment and then life was back on again* (...) *the doctor said I had leukemia, that the treatment would be long. I thought: it can’t be, and that’s when the shock began for me.* (P6)

*I discovered and investigated it later* [lymphoma], *I was anesthetized, I wanted to know about the disease later. At first it wasn’t something I wanted to know, I was researching more about chemotherapy, but it wasn’t something I was researching all the time.* (P4)

(...) *it was a shock to receive the news, so at first I wanted to keep to myself, to be quieter. I was very nervous, I heard about people who felt bad, I was very afraid of feeling like that.* (P12)

Other excerpts from speeches describe how adolescents and young people in rural areas are impacted when they become aware of the long duration of treatment. In view of this, some initially try not to know about the disease, isolate themselves, and demonstrate feelings such as nervousness and fear, indicating individual vulnerability.

*Losing contact: “I am your friend at a party, but I am not when you are sick”*

The participants’ statements show that losing contact with friends during cancer treatment is a challenging situation that adolescents and young people in rural areas face. These losses of meaningful connections reduce the social cycle.

*Because at the time of diagnosis we see who is really with us. I had a best friend, she doesn’t care about me, I called her, told her and now she doesn’t even pay attention to me* (...) *there are few who really stay, with time they abandon us* (...) *many moved, disappeared and some call me from time to time just to ask.* (P1)

*After the lymphoma diagnosis, many people distanced themselves. My social circle today is my family* (...) *I had some friends from school, but the friendship was no longer the same, the search was no longer the same. You know that story that at a party I’m your friend, but when you’re sick I’m not. They found out and didn’t actually come looking for me*. (P4)

(...) *if I make someone my friend, it’s friendship, it’s not “ah, let’s do something good” and when I end up in a hospital, I don’t have it.* (P3)

The speeches of adolescents and young people portrayed that, after being diagnosed with cancer, the friendships built before treatment did not remain the same. These impasses resulted in the distancing and abandonment of friends and the consequent loss of contact, revealing individual and social vulnerability.

**Figure 1:**
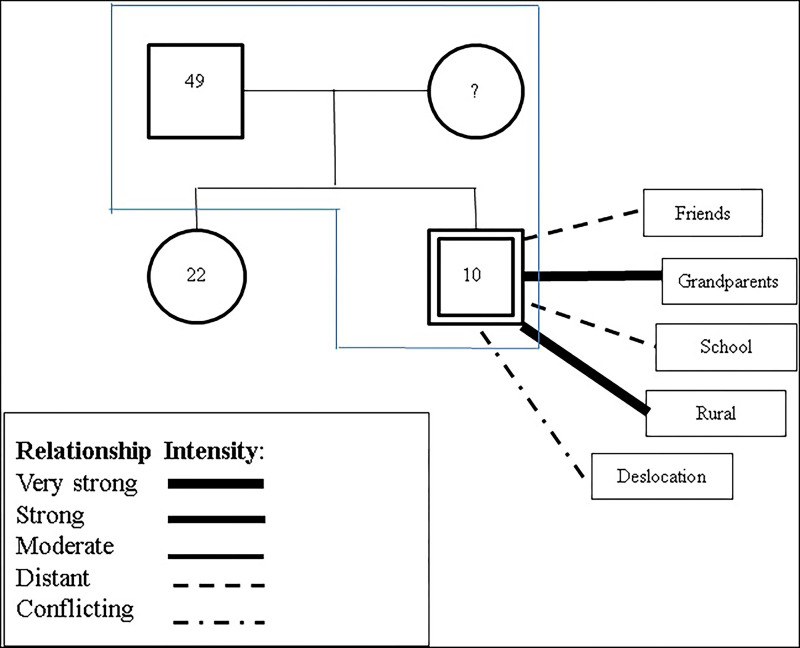
Genogram and Ecomap, Participant 15.

**Figure 2:**
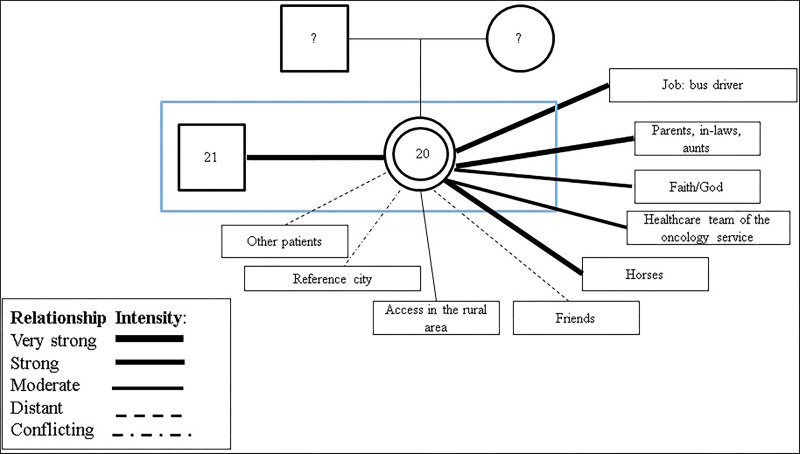
Genogram and Ecomap, Participant 4.

In the genogram and ecomap illustrated in Figure 1 and in Figure 2, social distancing between friends and school stands out, revealing a restricted social network after the cancer diagnosis. This may indicate a lack of social support and isolation. This phenomenon may be related to several factors, such as adaptation difficulties, communication problems or even emotional issues that hinder the construction of emotional bonds.

The conflicting relationship with the Municipality of reference and displacement can exacerbate feelings of abandonment or helplessness, negatively impacting the individual’s mental health and their willingness to seek and maintain care and causing dissatisfaction with health services and resistance to treatment, which can affect well-being and access to essential services.

*Limited reality: “I don’t live anymore, I live for the treatment”*

The journey of experiencing cancer treatment imposes limits on the way of life, routine, and social life in which adolescents and young people end up having to withdraw from their usual activities and leisure activities due to immunological instability, to avoid the risk of getting hurt and having to take extra care with the disease.

(...) *I started treatment when I was 14, I didn’t even have time to enjoy my youth, which is being able to go to a dance, play football, a game of bocce, snooker, you can’t do that with treatment.* (P9)

(...) *before I could do whatever I wanted, now I have to take care of my health and with low immunity, I can’t ride horses, ride motorbikes, bicycles, play football, there’s a risk of falling and getting hurt. And what I really wanted was to ride horses and motorbikes. In the summer I used to swim in the pool or in the river, but now I can’t because of the PICC* [catheter]. (P1)

*I spent a lot of time stuck in the hospital, the same thing every day, and when I went home I wanted to do other things, I wanted to enjoy myself, have freedom* (...) *I love playing in the mud, but I couldn’t, so my mother bought gloves for me to play in the mud.* (P12)

*This is something I wouldn’t wish on anyone, not even my worst enemy. I don’t live anymore, I virtually live for this, everything changes, your world turns around. I lived, went out, worked and then out of nowhere, I live for the treatment and do nothing else but the treatment*. (P11)

This new reality forces participants to live for treatment, having to stop not only their lives, but their process of being a teenager, limited from playing, and of being young, unable to work, go to dances or parties, and carry out other leisure activities outside the hospital setting. These situations encourage the development of individual and social vulnerability, as they miss doing the things they did before treatment and being able to work.

*I had some restrictions on what I could eat and drink. I saw other people eating and I couldn’t eat peanuts and drink a coke* (P10)

*The doctor said maintain normal life. But how normal, it’s not a normal life, not being able to eat honey, drink cow’s milk*. (P12)

Dietary restrictions were other similar dilemmas among adolescents and young people while undergoing treatment. The speeches discuss food desires and the limitation of seeing others and not being able to eat or drink. Limitations also occur in the final phase of treatment, that is, in maintenance, in which “normal life” is not stabilized and does not continue outside the context of the disease. This reality of “I couldn’t be” and “it’s not a normal life” exposes them to a vulnerability that is mainly individual.

### Access and Travel: Difficulties in Going for Treatment

The need for adolescents and young people to travel from rural areas to receive cancer treatment in the city is perceived as a tiring journey, as they have to travel a long way, for a long period of time, often having to get up early to receive treatment. The long distance impacts the desire to stop undergoing treatment. Adolescents and young people often end up traveling by their own means so that access to oncology services is quicker, even in the event of an oncological emergency. However, sometimes they could not drive due to the effects of the treatment, for fear of an accident happening and having to interrupt the treatment.

(...) *you go home and get a fever or something, you have to come running here, it’s far away. But sometimes we come in our own car, so we don’t have to stay up all night.* (P1)

(...) *I had to wake up early, it was complicated, I woke up at 3 am, I just fell into bed. I underwent chemotherapy every week, three times, there were days when I felt like quitting everything, abandoning these trips, we are already tired when I arrive and still have to undergo chemotherapy* (P5)

(...) *I had a way of getting around, but sometimes I couldn’t drive because of the treatment, I was afraid of an accident and having to interrupt the treatment, so I took the bus.* (P7)

(...) *the cars* [from the reference municipality] *were not good. The roads are not in good condition to get to the city. When the weather was bad and my immunity was low and I came to the city, I couldn’t take any risks there. (P4)*

*They* [drivers from the reference municipality] *came to pick me up, there were times when they even complained that they didn’t want to come, they said we would have to go to court. There was a time when they left me here and went away. I think a lot of that affected me, because I had to come here and there was no way.* (P10)

In their reports, participants express the difficulties they face in their home towns, such as few bus lines, poor roads and inadequate healthcare services. The young people mentioned that due to the effects of the treatment they could not drive to undergo it, which led to a problem in terms of travel and access to the oncology service. These situations signal individual, social and, above all, programmatic vulnerability.

## DISCUSSION

The situations of vulnerability faced by adolescents and young people in rural areas undergoing cancer treatment reveal a complex convergence of factors that go beyond individual health issues. The findings of this study reveal new and relevant data on the vulnerability faced by adolescents and young people from rural settings undergoing cancer treatment, indicating that they not only deal with direct treatment challenges, such as the side effects of chemotherapy and the emotional impact of the diagnosis, but also face barriers to access and travel to the hospital that aggravate their situation of vulnerability. In the first place, the emotional impact of a cancer diagnosis is one of the main challenges identified. The literature points out that this circumstance can be devastating, especially in phases of life in which the construction of identity, the search for autonomy and independence, and socialization are crucial. This can significantly influence how they interpret, think, and present themselves when faced with vulnerability to cancer. Additionally, adolescents and young adults may not understand what it really means to have cancer, which may be a strategy to avoid diagnosis^(17,18)^.

A cancer diagnosis raises doubts and demands among adolescents and young people living in rural areas, leading them to reflect on their lives and how treatment and its results can change them. This often leads to a reevaluation of values and goals and the need to realign behaviors to fight cancer and its effects for a relatively long time^(18)^. Adolescents and young people, because they do not experience milestones and experiences of adult life, end up feeling insecure and in internal conflict, there is a loss of identity and control of the situation in relation to the oncological disease, between the real - living with the disease and facing the oncological treatment and what they expected to have - a healthy life^(11)^.

This reality is consistent with the literature^(5)^, where an uncertain or poor cancer prognosis is a challenge for adolescents and young people. Challenges are ongoing, such as feeling inferior to their former self and others, feeling regret about life, and being unable to plan for the future. This can burden the lives of adolescents and young people in rural areas, disrupt their personal lives, and thus mark a group of vulnerable people. Therefore, one of the challenges of the cancer experience among adolescents and young people is the difficulty of experiencing the unknown^(5)^.

The distancing and abandonment of friends are noted in the experiences of adolescents and young people in rural contexts^(7)^. In this regard, a study carried out in the state of Minas Gerais, Brazil, showed that this situation causes resentment due to the absence of friends during treatment. Teenagers and young people try to justify this behavior of their friends due to emotional immaturity, realizing that they are experiencing another moment in life^(19)^. Furthermore, the separation of these bonds is a factor that can lead to social isolation among adolescents and young people and worsening of their mental health conditions.

Experiencing a reality limited by cancer is a phenomenon that was also observed in research carried out in the Netherlands^(20)^. Even if the treatment presents satisfactory results and the cancer is stabilized, living with the disease takes center stage and limitations can occur in all areas of their lives^(20)^. As a result of treatment, interaction with peers and family results in distancing from the social environment and recreational activities such as playing ball, riding a bike, horseback riding, going to a party and visiting friends, given that adolescents and young people undergoing cancer treatment are restricted to the hospital routine^(17,19)^.

This experience of breaking with the previous routine due to treatment triggers an individual vulnerability. This aspect of vulnerability is enhanced due to the health condition and when there is a lack of resources to assist in this process^(21)^. This causes a conflict about who the teenager and young adult used to be, while questioning their identity of who they are now. Many teenagers and young people do not know how to deal with limited reality and do not accept changes and express the desire to return to their old life^(7)^.

In this sense, the side effects of treatment and dietary restrictions are also critical aspects that impact the quality of life of adolescents and young people. Cancer treatment results in a new way of life trapped in a therapeutic routine that interrupts social interaction with friends and family, as well as daily activities, resulting in social vulnerability^(22)^. Social restriction can be considered one of the most difficult experiences of the disease, as adolescents and young people end up not being able to fully develop their adolescence and youth^(20)^.

During cancer treatment, adolescents and young people face difficulties in maintaining their usual eating habits, which creates embarrassment when adapting to new diets. Accustomed to a diet that respects their desires at home, they feel uncomfortable when they see other teenagers and young people consuming food they cannot eat, such as fried food, barbecue, soft drinks, and sweets. This dietary change requires them to make a significant effort to abstain from habits that were part of their lives before their cancer diagnosis^(18)^.

Time off work during cancer treatment requires adolescents and young people to make adjustments to their professional lives, producing negative feelings of uselessness and social disconnection. A Norwegian study found that 25% of young cancer survivors were not employed after treatment and 38% reported low productive capacity, associated with factors such as low education, depression, and poor self-rated health^(23)^. Furthermore, a Canadian cohort study found that nearly half of adolescent and young adult survivors experienced difficulties with concentration and memory, negatively impacting their employment prospects^(24)^. The literature suggests that, for the rural population, health is linked to the ability to study, work, maintain social relationships and independence^(5)^. This is reflected in programmatic vulnerability, highlighting the need for public policies that improve access and opportunities for adolescents and young people, promoting their well-being.

In this context, the lack of resources and recreational opportunities in rural areas limits social activities for adolescents and young people undergoing cancer treatment^(25)^. It is often at this time that they become more aware of their health condition, which is heightened by the restriction between periods of hospitalization and home care, increasing their individual vulnerability^(26)^.

Currently, access to cancer treatment for adolescents and young people in rural areas presents obstacles that increase their individual, social, and programmatic vulnerability^(8,27)^. This population experiences a therapeutic cycle that requires them to always be up to date with their treatment, constantly going to do it and returning home. This experience may result in greater psychosocial and physical distress compared to those living in urban areas, close to the treatment site^(25)^. The long, difficult distances and time away from home and the support and reassurance from their family can lead to early interruption of cancer treatment^(28)^.

The distance between the residence and the place of oncological treatment makes access to health care difficult for adolescents and young people, due to poor roads and adverse weather conditions. The lack of adequate transportation and financial resources for travel worsens this situation, leading them to depend on their own means to reach oncology services, which represents a programmatic vulnerability. However, having guaranteed convenient access to travel is not always an available reality. The literature highlights that equity in access to health services is a fundamental right^(28)^.

Rural areas are considered vulnerable, increasing the susceptibility of adolescents and young people to health risks due to isolation and lack of access to health services^(9)^. During the COVID-19 pandemic, adolescent and young adult cancer survivors in rural areas face disparities in cancer treatment and difficulties accessing care and social support, affecting their mental health^(29)^. Studies indicate that these adolescents and young people who live in rural areas need support to organize their travels and support homes can be an alternative to facilitate cancer treatment in urban centers^(11,28)^.

Within this framework, infrastructure and human resources for health care can vary substantially between rural and urban communities, with diagnostic and treatment procedures often centralized in urban areas^(29)^. Access to health services is challenging both in specialized centers and in primary health care. To optimize cancer treatment in adolescents and young people, collaboration within different levels of health is important, especially considering that access to social workers and mental health professionals is limited in rural areas^(28)^.

Based on the above, the different situations of individual, social, and programmatic vulnerability in the face of oncological treatment experienced by adolescents and young people in rural settings are evident. These differences are influenced by factors such as limited access to health information, which can lead to reduced awareness of the disease and delays in diagnosis^(20)^. Furthermore, stigma, unfavorable socioeconomic conditions, geographic isolation, and difficulties with transportation and access to educational resources aggravate these situations, hindering the search for health care and sharing of information about the disease^(19,25)^.

In this sense, Ayres defines vulnerability as a characteristic of groups susceptible to harm, resulting from the interaction between individuals, such as adolescents and young people, and their external environment^(9)^. Greater vulnerability is associated with increased morbidity and mortality and decreased quality of life, being more pronounced during transitions and major life changes^(27)^. The concept of vulnerability relates to risks and helplessness, resulting from exposure to stress and difficulties in managing these risks, involving external and internal factors that affect the ability to deal with adverse situations^(9)^.

The individual, social, and programmatic vulnerability of people with chronic diseases is a complex and critical public health issue. Ayres^(9)^ highlights that the lack of information about the disease increases susceptibility to isolation and helplessness, affecting not only physical health, but also individuals’ self-esteem, identity and future prospects.

Adolescents and young people from rural areas need to travel to urban centers to receive adequate treatment, face difficulties in understanding their diagnoses and limitations in local services^(4,29)^. However, it should be noted that there is a lack of public policies and specific health programs for this population, such as specialized health units and multidisciplinary teams.

To address situations of vulnerability, it is necessary to invest in education to promote awareness about cancer in rural areas, through prevention and information campaigns, aiming to reduce social stigma and promote early detection of the disease^(27)^. Ayres^(9)^ suggests the implementation of public policies aimed at reducing social inequalities and strengthening the social support network.

Vulnerability is a widely discussed topic in different contexts, given the immesurable number of uses and references to this word, in common sense and in political and scientific circles. Therefore, understanding vulnerability goes beyond a concept, it is an effort to understand the complexity of the experience of adolescents and young people in rural settings undergoing cancer treatment. The experience of cancer treatment can be intensified if they are unable to interpret their context and experience with cancer and act on this situation critically. In short, the situations of vulnerability faced by adolescents and young people from rural areas who are undergoing cancer treatment must be understood in an integrated manner, considering not only individual aspects, but also collective, contextual, cultural, economic, and political aspects. Appreciating these multiple factors is critical to developing effective interventions that promote health and well-being of these vulnerable populations.

A limitation of the study is the fact that it was developed in only one setting, a hospital, and in only one geographic region of Brazil. The implications for health practice reinforce the importance of nurses carrying out a comprehensive assessment of the individual situation of each rural adolescent or young person undergoing cancer treatment, considering not only their health condition, but also social, economic, and geographic factors. This study does not intend to exhaust this subject, but to collaborate in amplifying and stimulating new research and provoking reflections on the topic.

## CONCLUSION

Rural adolescents and young people undergoing cancer treatment face situations of vulnerability related to the disease, limited access to adequate health care, and social and emotional difficulties. A cancer diagnosis is the first emotional impact that leaves them susceptible to individual vulnerability as they do not expect to experience, for a long period, this process of illness in adolescence and youth. Losing contact with friends, which causes them to distance themselves, leads to negative feelings and isolation. The need to leave work limited social interaction, depriving them of professional opportunities important for their personal development.

Chemotherapy side effects and dietary restrictions also have a significant impact. Access and travel to receive treatment were seen as a challenge for rural adolescents and young people. They also face negative reactions from people in their social settings, fostering individual and social vulnerability.

The results of the study suggest the need to create listening and embracing spaces in oncology services, allowing adolescents and young people to share their experiences and needs. This can improve the quality of care and adherence to treatment. The study fills a gap in the literature on the health of adolescents and young people in rural areas, contributing to a deeper understanding of their needs and challenges. The findings can serve as a basis for new studies that explore other dimensions of the health and well-being of this population. The results can encourage social and community initiatives, such as support groups and social integration programs.
