# Supplementary material for: Vulnerability situations of adolescents and young people from rural areas undergoing cancer treatment
Source: Rev Esc Enferm USP. 2025 Feb 10;58:e20240250. doi: 10.1590/1980-220X-REEUSP-2024-0250en (PMC11823437; doi:10.1590/1980-220X-REEUSP-2024-0250en)
Supplement: Translation

# Translation: Situações de vulnerabilidade de adolescentes e jovens do contexto rural em tratamento oncológico*

**DOI:** 10.1590/1980-220X-REEUSP-2024-0250pt

**Authors:** Fernanda Duarte Siqueira, Graciela Dutra Sehnem, Nara Marilene Oliveira Girardon-Perlini, Aline Cammarano Ribeiro, Cíntia Beatriz Goi, Camila Lovato de Figueiredo, Eliane Tatsch Neves

**Affiliations:** 1 Universidade Federal de Santa Maria, Departamento de Enfermagem, Santa Maria, RS, Brasil.; 2 Universidade Federal de Santa Maria, Programa de Pós-Graduação em Enfermagem, Santa Maria, RS, Brasil.

**Keywords:** Adolescente, Oncologia, População Rural, Vulnerabilidade em Saúde

## Abstract

**Objetivo:**

Compreender as situações de vulnerabilidade de adolescentes e jovens do contexto rural em tratamento oncológico.

**Método:**

Pesquisa qualitativa, mediada pelo referencial da vulnerabilidade em saúde, desenvolvida em um serviço de oncologia no Sul do Brasil com adolescentes e jovens do meio rural em tratamento oncológico, por meio de entrevistas semiestruturadas, audiogravadas, de abril de 2021 a setembro de 2022, ocorrida em cinco fases, com análise dos dados realizada à luz da Análise Temática Indutiva Reflexiva.

**Resultados:**

Participaram do estudo 11 adolescentes e quatro jovens, a maioria do sexo masculino, solteiros, com renda familiar de até três salários mínimos e diagnóstico de Leucemia Linfoblástica Aguda. As situações de vulnerabilidade dos adolescentes e jovens do contexto rural em tratamento oncológico envolvem desafios, como o impacto emocional do diagnóstico, a perda do contato com os amigos, efeitos colaterais da quimioterapia, as restrições alimentares, o acesso e o deslocamento para realizar o tratamento.

**Conclusão:**

Os adolescentes e jovens em tratamento oncológico enfrentam situações de vulnerabilidade relacionadas à doença, limitações de acesso a cuidados de saúde adequados, dificuldades sociais e emocionais.

## INTRODUÇÃO

A adolescência e juventude correspondem a processos biológicos, construções culturais, político-econômicas e territoriais que ao longo de diferentes épocas e processos históricos foram adquirindo diferentes significados e concepções^(1)^. A Organização Mundial da Saúde (OMS) faz referência aos termos adolescentes e jovens, cuja faixa etária compreende pessoas de 10 a 24 anos^(2)^, definição esta utilizada na construção deste estudo. Nessas fases, o tratamento oncológico pode interferir no processo de formação, desenvolvimento e amadurecimento, produzindo repercussões no cotidiano e nas atividades de lazer^(3)^.

No Brasil, o câncer já representa a principal causa de morte por doença entre pessoas com 19 anos ou menos. Aproximadamente 6% de todos os cânceres acometem adolescentes e jovens entre 15 e 29 anos. Considerando esse panorama, 80% de crianças, adolescentes e jovens podem ser curadas, se diagnosticados precocemente e realizarem tratamento adequado em centros especializados^(4)^.

Nesse cenário, a realidade do indivíduo em tratamento oncológico pode ser influenciada pelo contexto sociocultural, econômico e de vida dos envolvidos, apresentando particularidades que podem estar relacionadas ao fato de residirem em áreas urbanas ou rurais^(5)^. Nesse sentido, o meio rural pode ser compreendido como uma forma de vida que abrange todos os membros que ali vivem e trabalham^(6)^. Viver nesse meio apresenta desafios específicos como, por exemplo, a falta de infraestrutura adequada, estradas e transporte público, dificultando o acesso a serviços de saúde, impactando na vida dos adolescentes e jovens e podendo agravar sua condição de saúde no tratamento oncológico^(7)^. Estudo que investigou as disparidades rural-urbanas mostrou que em áreas rurais a detecção precoce e o acesso ao tratamento oncológico é um desafio, resultando em piores desfechos de saúde^(4)^.

O crescimento e o desenvolvimento dos adolescentes e dos jovens brasileiros, muitas vezes, são permeados por um contexto de vulnerabilidade como a pobreza, as infecções sexualmente transmissíveis, as doenças crônicas, entre outros^(8)^. Nesse âmbito, a vulnerabilidade resume-se à percepção ampliada e reflexiva que considera a chance de exposição das pessoas ao adoecimento e os consequentes impactos em totalidades dinâmicas, passando por um conjunto de aspectos não apenas individuais, mas coletivos, contextuais, culturais, econômicos e políticos que acarretam suscetibilidade ao adoecimento^(9)^.

Para tanto, a vulnerabilidade envolve a articulação de três componentes: o individual, o social e o programático. O individual refere-se ao grau e à qualidade da informação que os indivíduos dispõem sobre o problema e à possibilidade de empregá-las para a proteção. O social abrange aspectos que dependem do acesso aos meios de comunicação, disponibilidade de recursos materiais e fatores políticos. Já o programático tem relação com as ações de programas destinados à prevenção e ao cuidado, podendo abarcar políticas locais, regionais e nacionais que devem ser disponibilizadas de modo efetivo e democrático^(9)^.

A Enfermagem alia-se a esse contexto como profissão com papel importante no desenvolvimento de ações de cuidado aos adolescentes e jovens, tendo em vista sua atuação na prevenção e promoção da saúde, no cuidado e no tratamento oncológico^(10)^. Desse modo, os profissionais de saúde, em especial o enfermeiro, estão na linha de frente dos serviços de saúde e necessitam considerar as situações de vulnerabilidade e as características dos cenários em que estas pessoas estão inseridas^(11)^.

Estudo de revisão que objetivou identificar e caracterizar as tendências da produção científica brasileira acerca da saúde de adolescentes e jovens vivendo no contexto rural ressaltou a lacuna existente no conhecimento nesta área. As evidências revelaram que a temática é ainda incipiente, com poucos estudos em que os principais temas abordados foram relacionados a aspectos de risco à saúde, hábitos e estilos de vida prejudiciais, como alimentação inadequada, associados às doenças crônicas não transmissíveis. Além disso, foram abordados condicionantes de saúde, atividade física, necessidades de promoção da saúde, acesso e utilização de serviços, além de perspectivas culturais, de gênero, sexualidade, reprodução e violência^(11)^, o que justifica a importância de aprofundar a investigação nestes tópicos para melhor compreender e atender as necessidades desse público. A partir disso, questionou-se: quais as situações de vulnerabilidade que adolescentes e jovens do contexto rural em tratamento oncológico vivenciam? Assim, o objetivo deste estudo foi compreender as situações de vulnerabilidade de adolescentes e jovens do contexto rural em tratamento oncológico.

## MÉTODO

### Tipo de Estudo

Estudo qualitativo, descritivo e exploratório^(12)^ que atende às recomendações do guia *Consolidated criteria for reporting qualitative research* (COREQ), versão em Português^(13)^ e é mediado pelo referencial da vulnerabilidade em saúde^(9)^.

### Cenário Do Estudo

O estudo foi realizado em um serviço de oncologia de um hospital escola, geral e público do Sul do Brasil.

### Participantes e Critérios de Seleção

Foram selecionados para o estudo adolescentes com idade entre 10 e 19 anos e jovens com idade até 24 anos conforme definições da OMS^(14)^, que estivessem em tratamento oncológico ou em acompanhamento clínico por no máximo cinco anos (após esse período, a pessoa pode ser considerada curada e sobrevivente do câncer)^(15)^, independentemente do tipo de câncer, que residissem no contexto rural no momento do diagnóstico. Dentre a população, foram excluídos os que não tivessem condições clínicas para participar do estudo de acordo com a indicação da equipe do serviço de oncologia.

### Coleta de Dados

A coleta de dados ocorreu presencialmente no período de abril de 2021 a setembro de 2022. O estudo foi desenvolvido em cinco fases. Na primeira, foi realizada a aproximação com os profissionais dos serviços, sendo apresentada a proposta da pesquisa à equipe e a importância em desenvolvê-la, bem como o reconhecimento e familiarização da rotina do campo de estudo, acompanhamento do tratamento oncológico e estabelecimento de vínculo. A rotina dos adolescentes e jovens em tratamento oncológico variava dependendo do tipo de câncer, estágio da doença e do plano de tratamento específico.

Na segunda fase ocorreu o recrutamento dos participantes, em que se utilizou a listagem dos pacientes hospitalizados e a agenda semanal de atendimento. Além disso, foi realizado o levantamento de dados em prontuário dos adolescentes e jovens, via Aplicativo de Gestão para Hospitais Universitários (AGHU), acessando as consultas agendadas para selecionar os participantes e coletar dados de caracterização sociodemográfica como: sexo, idade, situação conjugal, procedência, endereço, escolaridade, se está indo à escola, se está trabalhando, renda familiar, diagnóstico médico, tempo de tratamento, data do início do tratamento, tratamento proposto, situação do tratamento, recidiva e deslocamento. Para tanto, utilizou-se a amostragem intencional, sendo recrutados todos os adolescentes e jovens que residiam no contexto rural.

Posteriormente, na terceira fase, foi solicitado aos profissionais dos serviços de oncologia auxílio em estabelecer o primeiro contato entre os adolescentes, os jovens e a pesquisadora, informando-os sobre o estudo e/ou indicando os possíveis participantes. Na quarta fase, após identificação dos participantes que atendiam aos critérios de seleção, foi estabelecido o contato pessoalmente entre a pesquisadora, o adolescente e/ou jovem e seu familiar ou responsável legal durante a hospitalização, ou no dia do agendamento do tratamento quimioterápico previsto ou da consulta médica. Em consonância, foi realizado o convite para participar do estudo. Na quinta fase, a pesquisa prosseguiu por meio da entrevista face a face, com roteiro semiestruturado, guiado pelas seguintes questões norteadoras: “Como é para você fazer o tratamento?”; “Como você descobriu a sua doença?”; “Quem tem te ajudado durante o tratamento?”; “Como ocorre o teu deslocamento para a rotina de tratamento?”; “Como o tratamento influencia em seu lazer, na escola, trabalho, com amigos e familiares?”. As entrevistas foram realizadas, apenas, com a presença da pesquisadora e do participante, momento em que foi construído o genograma e ecomapa, utilizados como ferramentas de aproximação com os participantes. As entrevistas foram interrompidas quando os dados foram saturados.

As entrevistas foram audiogravadas, ocorreram antes e durante a infusão da quimioterapia ou após a consulta médica, todas no período da manhã, e apresentaram uma duração média de 52 minutos em uma sala ou espaço reservado disponível e de preferência dos adolescentes e jovens. Não houve desistências após o aceite em participar da entrevista. Como a coleta de dados ocorreu em meio à pandemia da COVID-19, as medidas de biossegurança e prevenção de contaminação foram seguidas pela pesquisadora e entrevistados.

### Análise e Tratamento Dos Dados

As entrevistas foram transcritas, organizadas e submetidas a análise Temática Indutiva Reflexiva proposta por Braun e Clarke (2019)^(16)^, constituída por seis fases: 1) Familiarizando-se com seus dados; 2) Gerando códigos iniciais; 3) Buscando por temas, agrupando os códigos em temas potenciais, reunindo todos os dados relevantes para cada tema potencial; 4) Revisando temas identificados; 5) Definindo e nomeando temas; 6) Produzindo o relatório. Dessa forma, os dados foram produzidos e agrupados ao redor de uma categoria temática e quatro subtemas destacados e apresentados nos resultados.

Como o objetivo do presente estudo foi compreender o fenômeno do tratamento oncológico, a despeito da diferença de faixa etária entre os participantes com menos idade e os considerados “mais velhos”, as situações de vulnerabilidade puderam ser identificadas, considerando-se a homogeneidade e a heterogeneidade tanto entre os adolescentes quanto os jovens do contexto rural.

### Aspectos Éticos

O estudo seguiu todas as diretrizes e prerrogativas legais estabelecidas pelas Resoluções n.º 466/ 2012 e 510/2016, do Conselho Nacional de Saúde. Obteve aprovação do Comitê de Ética em Pesquisa (CEP) sob o registro 4.594.079. Todos os participantes foram informados em linguagem clara e objetiva a respeito da natureza da pesquisa, seus objetivos, a maneira como proceder para responder às entrevistas. Entregue o Termo de Consentimento Livre e Esclarecido (TCLE) para os adolescentes e jovens maiores de 18 anos e o Termo de Assentimento para os participantes menores de 18 anos condicionados à assinatura dos pais ou responsável legal, em duas vias, ficando uma para o participante e a outra para a pesquisadora. O anonimato dos participantes do estudo foi preservado por meio da adoção de códigos, substituindo-se seus nomes pela letra P, na ordem na qual a entrevista foi realizada (P1, P2...P15).

## RESULTADOS

Participaram do estudo 11 (73,3%) adolescentes e quatro jovens (26,7%), com idade média de 15,6 anos. Entre os adolescentes as idades variaram de 10 a 19 anos e nos jovens entre 20 a 22 anos. Dentre os adolescentes, sete (63,7%) eram do sexo masculino e quatro (36,3%) do sexo feminino. No público jovem, três (75%) eram do sexo masculino e uma (25%) participante do sexo feminino. Assim, pode-se resumir que 10 (66,7%) participantes eram do sexo masculino e cinco (33,3%) do sexo feminino. Os participantes eram procedentes de 15 localidades rurais de 13 municípios do Estado do Rio Grande do Sul, predominando os da região centro-oeste do Estado.

Em relação à situação conjugal, 10 (66,7%) declaram-se solteiros, três (20%) estar namorando e dois (13,3%) casados. Quanto à escolaridade, cinco (33,3%) apresentaram ensino médio completo, cinco (33,3%) ensino fundamental incompleto, três (20%) ensino médio incompleto e dois (13,3%) ensino fundamental completo. No que se refere às atividades escolares, entre os adolescentes, seis (54,5%) interromperam os estudos, durante o tratamento, três (27,3%) estavam participando remotamente e dois (18,2%) frequentavam a escola. Dos participantes jovens, dois (50%) trancaram a faculdade, de administração e zootecnia. Entre os adolescentes e jovens, sete (46,7%) exerciam atividade remunerada e todos pararam de trabalhar, devido ao tratamento.

Nesse contexto, pode-se verificar que a renda familiar foi de até três salários-mínimos. No que tange ao diagnóstico médico, identificou-se que nove (60%) dos participantes apresentaram diagnóstico de Leucemia Linfoblástica Aguda, quatro (26,7%) Leucemia Mieloide Aguda, um Linfoma de Hodgkin (6,7%) e um (6,7%) Linfoma não Hodgkin. Quanto ao tratamento, predominou a utilização da quimioterapia. Em relação à situação do tratamento, oito (53,3%) estavam em acompanhamento clínico, dois (13,3%) na fase de indução, dois (13,3%) na fase de manutenção, um (6,7%) na fase de consolidação, um (6,7%) em tratamento para transplante e um (6,7%) em avaliação diagnóstica por recidiva. Ressalta-se que em três participantes ocorreu recidiva. O tempo de tratamento variou de três a 60 meses.

A maioria (46,7%) dos participantes deslocou-se para realizar o tratamento oncológico utilizando o transporte da Secretária Municipal de Saúde de seu município de referência, cinco (33,3%) deslocaram-se por meios próprios (carro ou ônibus) e três (20%) participantes revezavam o deslocamento entre o transporte da Secretária Municipal de saúde e meio próprio.

A partir da análise dos resultados identificaram-se situações de vulnerabilidade vivenciadas pelos participantes a partir de desafios do tratamento oncológico, constituindo-se na seguinte categoria temática: “Desafios do tratamento oncológico para o adolescente e jovem do contexto rural”.

Os adolescentes e jovens do contexto rural vivenciam a experiência do tratamento oncológico, enfrentando diversos desafios desde o momento da descoberta do câncer até os diversos percalços envolvidos, o que os predispõem a vulnerabilidade individual, social e programática. Desvelou-se a ocorrência de ruptura no cotidiano, nova realidade desconhecida, restritiva e duradoura, desajustes nos vínculos afetivos, impactos negativos frente aos efeitos colaterais do tratamento oncológico e dificuldades de acesso.

*Choque do desconhecido: “não sabia que leucemia era um câncer”*

As narrativas dos adolescentes e jovens deste estudo revelam que a descoberta do câncer é permeada pelo desconhecimento ou informação estigmatizada, associada à representação social da doença, produzindo sensações de medo, preocupações até mesmo de morte, sinalizando uma vulnerabilidade individual, conforme relatadas nas falas a seguir:

(...) *não me falaram que eu estava com câncer, falaram que era leucemia. Eu não sabia que leucemia era um câncer. O médico disse “você está com uma anemia muito forte e pode virar uma leucemia”. Daí minha irmã que estava comigo começou a chorar e eu não entendi nada, e falei “por que está chorando, eu só vou tomar um soro e vou embora”*. (P3)

(...) *eu não sabia o que era* (...) *eu não sabia o que estava acontecendo direito, nunca ouvi falar, no início pensei que era alguma coisa muito ruim e foi muito ruim* (...) *era uma situação de morte. Eu não podia escovar meus dentes que saia sangue, eu achei que ia morrer.* (P10)

(...) *foi um “boom” quando a médica falou para o pai o que eu tinha. Eu sabia que leucemia não era bom, mas não imaginei que era um câncer. Fiquei com medo, sem saber o que esperar. E para mim, leucemia era uma coisinha no sangue, eu não dei muita bola. Parece que a hora que tu descobre desanda tudo, fui de mal a pior.* (P11)

Essas situações, provenientes das falas, ocorrem em razão de eles não reconhecerem, ou não terem sido informados, ou esclarecidos sobre sua atual condição, ou por não compreenderem a magnitude de sua doença, ou não terem o entendimento de como poderia ocorrer o tratamento e não esperarem vivenciar esse processo de adoecimento, durante a adolescência e juventude.

(...) *a hora que ele* [médico] *falou que o tratamento não é curto é longo, daí pesou*. (P5)

*Foi um choque, na verdade, estava levando como se fosse uma gripe, um tratamento de dois, três meses e pronto segue a vida de novo* (...) *o doutor falou que eu estava com leucemia, que o tratamento era longo. Eu pensei: não pode ser, aí começou o choque para mim*. (P6)

*Fui descobrir e investigar depois* [linfoma], *eu estava anestesiada, quis saber da doença depois. No primeiro momento não foi algo que eu queria saber, pesquisava mais da quimioterapia, mas não era algo que estava toda hora pesquisando.* (P4)

(...) *dava aquele choque, de receber a notícia, por isso no início eu queria ficar mais na minha, mais quieta. Fiquei muito nervosa, eu ouvia falar de pessoas que ficam mal, eu tinha muito medo de ficar assim.* (P12)

Outros trechos de falas descrevem que os adolescentes e jovens do contexto rural ficam impactados, quando adquirem consciência do longo tempo de duração do tratamento. Diante disso, uns buscam no início não saber sobre a doença, isolam-se e demonstram sentimentos como nervosismo e medo, assinalando uma vulnerabilidade individual.

*Perdendo o contato: “na festa sou teu amigo, mas na doença não sou”*

As falas dos participantes discorrem que perder o contato com os amigos, durante o tratamento do câncer, é uma situação desafiadora que os adolescentes e jovens do contexto rural enfrentam. Estas perdas de conexões significativas reduzem o ciclo social.

*Porque a gente vê na hora do diagnóstico quem está com a gente de verdade. Eu tinha uma melhor amiga, está nem aí para mim, eu chamei ela, contei e agora ela nem bola me dá* (...) *são poucos que ficam mesmo, com o tempo vão abandonando* (...) *muitos mudaram, sumiram e alguns me chamam lá de vez em quando só para perguntar.* (P1)

*Depois do diagnóstico do linfoma muita gente se afastou. O meu meio social hoje é a minha família* (...) e*u tinha umas amigas de colégio, mas a amizade não ficou mais a mesma, a procura não foi mais a mesma. Sabe aquela história que na festa sou teu amigo, mas na doença não sou. Ficaram sabendo e na realidade não vieram me procurar.* (P4)

(...) *se tiro uma pessoa para amizade é amizade, não é “ah vamos fazer alguma coisa boa” e quando paro num hospital não tenho.* (P3)

Os discursos dos adolescentes e jovens retrataram que diante do diagnóstico do câncer, as amizades construídas antes do tratamento não se mantiveram as mesmas. Esses impasses repercutiram no afastamento e abandono dos amigos e consequente perda de contato, enunciando uma vulnerabilidade individual e social.

No genograma e ecomapa ilustrados na Figura 1 e Figura 2, destaca-se o distanciamento social entre amigos e escola, revelando uma rede social restrita após o diagnóstico de câncer. Isso pode indicar uma falta de suporte social e isolamento. Esse fenômeno pode estar relacionado a vários fatores, como dificuldades de adaptação, problemas de comunicação ou até questões emocionais que dificultam a formação de laços afetivos.

**Figura 1:** Genograma e Ecomapa, Participante 15.

**Figura 2:** Genograma e Ecomapa, Participante 4.

A relação conflitante com o Município de referência e deslocamento pode exacerbar sentimentos de abandono ou desamparo, impactando negativamente a saúde mental do indivíduo e sua disposição para buscar e manter cuidados e provocar insatisfação com os serviços de saúde e resistência ao tratamento, podendo afetar o bem-estar e o acesso a serviços essenciais.

*Realidade limitada: “eu não vivo mais, eu vivo para o tratamento”*

A trajetória de vivenciar o tratamento oncológico impõe limites ao modo de viver, na rotina e na vida social nos quais os adolescentes e jovens acabam tendo que se afastarem de suas atividades habituais e de lazer em decorrência da instabilidade imunológica, para não correr o risco de se machucarem e dos cuidados redobrados com a doença.

(...) *comecei com 14 anos o tratamento, nem deu tempo de aproveitar a juventude que é poder ir a um baile, jogar futebol, uma partida de bocha, sinuca, tu não consegue fazer isso pelo tratamento.* (P9)

(...) *antes podia fazer o que eu queria, agora tenho que ficar cuidando da saúde e com a imunidade baixa, não pode andar a cavalo, andar de moto, bicicleta, jogar bola, corre o risco de cair e se machucar. E, o que mais eu queria mesmo é andar a cavalo e de moto. Eu tomava no verão banho de piscina, de sanga, agora não pode pelo PIC* [cateter]. (P1)

*Eu ficava muito tempo presa no hospital, aquela mesma coisa todo dia e quando eu ia para casa já queria fazer outras coisas, eu queria aproveitar, ter liberdade* (...) *adoro brincar no barro, só que eu não podia, aí a mãe comprou luva para eu brincar no barro.* (P12)

*Isso aqui é uma coisa que eu não desejo para ninguém, nem para meu pior inimigo. Eu não vivo mais, eu vivo para isso aqui praticamente, muda tudo, teu mundo vira. Eu vivia, saía, trabalhava e aí do nada vira, vive para o tratamento e não faz outra coisa a não ser o tratamento.* (P11)

Essa nova realidade direciona os participantes a viverem para o tratamento, tendo que parar não somente sua vida, mas o seu processo de ser adolescente, limitado de brincar, e o de ser jovem, impossibilitado de trabalhar, sair em bailes ou festas e realizar outras atividades de lazer fora do contexto hospitalar. Essas situações instigam o desenvolvimento de vulnerabilidade individual e social, pois sentem falta de fazer as coisas que faziam antes do tratamento e de poder trabalhar.

*Eu tinha umas restrições do que eu podia comer e beber. Eu via as outras pessoas comendo e eu não podia comer amendoim e tomar uma coca* (P10)

*O médico disse manutenção vida normal. Mas como normal, não é uma vida normal, não poder comer mel, tomar leite de vaca*. (P12)

As restrições alimentares foram outros dilemas semelhantes entre os adolescentes e jovens durante o enfrentamento do tratamento. As falas discorrem os desejos alimentares e a limitação de ver os outros e não poder comer ou beber. As limitações ocorrem também na fase final do tratamento, ou seja, na manutenção, na qual a “vida normal” não é estabilizada e não segue fora do contexto da doença. Essa realidade do “não podia” e “não é uma vida normal” expõe-nos a uma vulnerabilidade principalmente individual.

### Acesso e Deslocamento: Dificuldades de ir Fazer o Tratamento

A necessidade dos adolescentes e jovens de se deslocarem do meio rural para o tratamento oncológico na cidade é percebida como uma viagem cansativa, por ter que percorrer um longo caminho, por um longo período, precisando, muitas vezes, madrugar para fazer o tratamento. A longa distância impacta na vontade de deixar de fazer o tratamento. Os adolescentes e jovens, muitas vezes, acabam deslocando-se por meios próprios para que o acesso ao serviço de oncologia seja mais rápido, até mesmo na ocorrência de uma emergência oncológica. Porém, às vezes, eles não podiam dirigir por efeito do tratamento, por medo de acontecer algum acidente e ter que interromper o tratamento.

(...) *vai para casa e dá uma febre ou outra coisa, tem que vir correndo para cá, é longinho. Mas, às vezes, a gente vem de carro próprio, para não ter que fazer uma madrugada.* (P1)

(...) t*inha que acordar cedo, era complicado, 3h da manhã eu acordava, só caía na cama. Eu fazia quimioterapia toda semana, três vezes, tinham dias que dava vontade de largar tudo, abandonar essas viagens, o cara já chega cansado e ainda tem que fazer quimioterapia* (P5)

(...) *eu tinha meio da locomoção, mas, às vezes, não podia dirigir por causa do tratamento, eu tinha medo de algum acidente e ter que interromper o tratamento, então eu pegava o ônibus*. (P7)

(...) *os carros* [do município de referência] *não eram bons. As estradas não têm muitas condições para chegar até a cidade. Quando o tempo estava ruim e eu com a imunidade baixa e vinha para cidade, não dava para correr risco lá*. (P4)

*Eles* [motorista do município de referência] v*inham me buscar, tinham vezes que até reclamavam que não queriam vir, diziam que ia ter que entrar na justiça. Teve uma vez que me deixaram aqui e foram embora. Acredito que uma boa parte disso me afetou, porque eu tinha que vir para cá e não tinha como.* (P10)

Nos relatos, os participantes manifestam as dificuldades que enfrentam nos seus municípios de origem, como poucas linhas de ônibus, estradas precárias e inadequado atendimento do serviço de saúde. Os jovens mencionaram que em virtude dos efeitos do tratamento não podiam dirigir para realizá-lo, o que promoveu o impasse de deslocamento e acesso até o serviço de oncologia. Essas situações sinalizam uma vulnerabilidade individual, social e principalmente programática.

## DISCUSSÃO

As situações de vulnerabilidade enfrentadas pelos adolescentes e jovens do contexto rural em tratamento oncológico revelam uma complexa convergência de fatores que vão além de questões individuais de saúde. Os achados do presente estudo revelam dados novos e relevantes sobre a vulnerabilidade enfrentada por adolescentes e jovens do contexto rural em tratamento oncológico, indicando que eles não apenas lidam com desafios diretos do tratamento, como os efeitos colaterais da quimioterapia e o impacto emocional do diagnóstico, mas também enfrentam barreiras no acesso e deslocamento que agravam sua situação de vulnerabilidade. Primeiramente, o impacto emocional do diagnóstico de câncer é um dos principais desafios identificados. A literatura aponta que essa circunstância pode ser devastadora, especialmente em fases da vida em que a construção da identidade, busca por autonomia e independência e a socialização são cruciais. Isto pode influenciar, significativamente, como eles interpretam, pensam e se apresentam frente à vulnerabilidade ao câncer. Além disso, os adolescentes e jovens podem não compreender o que realmente é ter câncer, o que pode ser uma estratégia para evitar o diagnóstico^(17,18)^.

O diagnóstico de câncer levanta dúvidas e demandas entre os adolescentes e jovens que vivem em áreas rurais, levando-os a refletir sobre as suas vidas e como o tratamento e os seus resultados podem mudá-las. Isso, muitas vezes, leva a uma reavaliação de valores e objetivos e à necessidade de realinhar comportamentos para combater o câncer e seus efeitos por um tempo relativamente longo^(18)^. Os adolescentes e jovens, por não vivenciarem marcos e experiências da vida adulta, acabam se sentindo inseguros e em conflito interno, ocorre a perda da identidade e do controle da situação em relação à doença oncológica, entre o real - viver com a doença e enfrentar o tratamento oncológico e o que esperava ter - vida sadia^(11)^.

Esta realidade é consistente com a literatura^(5)^, em que um prognóstico incerto ou ruim de câncer é um desafio para os adolescentes e jovens. Os desafios são contínuos, como sentir-se inferior ao seu eu anterior e aos outros, sentir um pesar pela vida e ser incapaz de planejar o futuro. Isso pode sobrecarregar a vida de adolescentes e jovens do contexto rural, atrapalhar sua vida pessoal, marcando assim um grupo de pessoas vulneráveis. Portanto, um dos desafios da experiência do câncer entre adolescentes e jovens é a dificuldade de vivenciar o desconhecido^(5)^.

O afastamento e abandono dos amigos são notados nas vivências dos adolescentes e jovens do contexto rural^(7)^. A esse respeito, estudo realizado no estado de Minas Gerais, Brasil, evidenciou que essa situação provoca ressentimento em virtude da ausência de amigos durante o tratamento. Adolescentes e jovens tentam justificar esse comportamento de amigos pela imaturidade emocional, percebendo que eles estão vivenciando outro momento de vida^(19)^. Além disso, o afastamento desses vínculos é um fator que pode levar ao isolamento social de adolescentes e jovens e um agravamento da saúde mental.

Vivenciar a realidade limitada pelo câncer é um fenômeno que também foi observado em uma pesquisa desenvolvida na Holanda^(20)^. Mesmo que o tratamento apresente resultados satisfatórios e o câncer esteja estabilizado, o viver a doença fica em primeiro plano e as limitações podem ocorrer em todas as áreas de suas vidas^(20)^. Em decorrência do tratamento, a convivência com pares e família resulta em um distanciamento do ambiente social e das atividades recreativas como jogar bola, andar de bicicleta, andar a cavalo, ir a uma festa e visitar amigos, tendo em vista que os adolescentes e jovens em tratamento oncológico ficam restritos à rotina hospitalar^(17,19)^.

Essa experiência de romper com a rotina anterior em função do tratamento desencadeia uma vulnerabilidade individual. Esse aspecto da vulnerabilidade potencializa-se, devido à condição de saúde e quando há falta de recursos para auxiliar nesse processo^(21)^. Isso provoca um conflito sobre quem o adolescente e o jovem costumava ser, enquanto questiona sua identidade de quem é agora. Muitos adolescentes e jovens não sabem lidar com a realidade limitada e não aceitam as mudanças e manifestam o desejo de voltar à sua antiga vida^(7)^.

Nessa direção, os efeitos colaterais do tratamento e as restrições alimentares também são aspectos críticos que impactam a qualidade de vida dos adolescentes e jovens. O tratamento oncológico resulta em um novo viver aprisionado a uma rotina terapêutica que interrompe a interação social, com amigos e familiares, além das atividades cotidianas, refletindo em uma vulnerabilidade social^(22)^. A restrição social pode ser considerada uma das experiências mais difíceis da doença, pois os adolescentes e jovens acabam não conseguindo desenvolver plenamente a adolescência e juventude^(20)^.

Durante o tratamento oncológico, adolescentes e jovens enfrentam dificuldades em manter seus hábitos alimentares habituais, o que gera constrangimentos ao se adaptarem a novas dietas. Acostumados a uma alimentação que respeita seus desejos em casa, eles se sentem incomodados ao ver outros adolescentes e jovens consumindo alimentos que não podem, como frituras, churrasco, refrigerantes e doces. Essa mudança alimentar exige deles um esforço significativo para se abster de hábitos que faziam parte de suas vidas antes do diagnóstico de câncer^(18)^.

O afastamento do trabalho, durante o tratamento oncológico, exige dos adolescentes e jovens ajustes nas suas vidas profissionais, produzindo sentimentos negativos de inutilidade e desconexão social. Um estudo norueguês revelou que 25% dos jovens sobreviventes de câncer não estavam empregados após o tratamento e 38% relataram baixa capacidade produtiva, associada a fatores como baixa escolaridade, depressão e autoavaliação de saúde precária^(23)^. Além disso, um estudo de coorte canadense evidenciou que quase metade dos adolescentes e jovens sobreviventes enfrentaram dificuldades de concentração e memória, impactando negativamente suas perspectivas de emprego^(24)^. A literatura sugere que, para a população rural, a saúde está ligada à capacidade de estudar, trabalhar, manter relações sociais e a independência^(5)^. Isto reflete na vulnerabilidade programática, destacando a necessidade de políticas públicas que melhorem o acesso e as oportunidades para os adolescentes e jovens, promovendo seu bem-estar.

Nesse contexto, a falta de recursos e oportunidades recreativas nas áreas rurais limita as atividades sociais para adolescentes e jovens em tratamento oncológico^(25)^. Muitas vezes, é nesse momento que eles se tornam mais conscientes de sua condição de saúde, o que é intensificado pela restrição entre os períodos de hospitalização e domicílio, aumentando sua vulnerabilidade individual^(26)^.

Atualmente, o acesso ao tratamento oncológico para adolescentes e jovens em áreas rurais apresenta obstáculos que aumentam sua vulnerabilidade individual, social e programática^(8,27)^. Essa população vivencia um ciclo terapêutico que exige que estejam sempre em dia com o tratamento, num constante ir fazê-lo e voltar para casa. Essa experiência pode resultar em maior sofrimento psicossocial e físico em comparação com aqueles que vivem em áreas urbanas, próximos ao local de tratamento^(25)^. As longas e difíceis distâncias e o tempo longe de casa e do apoio e conforto de sua família podem levar à interrupção precoce do tratamento oncológico^(28)^.

A distância entre a residência e o local de tratamento oncológico dificulta o acesso a cuidados de saúde para adolescentes e jovens, devido à precariedade de estradas e condições climáticas adversas. A falta de transporte adequado e recursos financeiros para o deslocamento agrava essa situação, levando-os a depender de meios próprios para chegar aos serviços de oncologia, o que representa uma vulnerabilidade programática. No entanto, ter acesso conveniente garantido para o deslocamento nem sempre é uma realidade disponível. A literatura destaca que a equidade no acesso aos serviços de saúde é um direito fundamental^(28)^.

As áreas rurais são consideradas vulneráveis, aumentando a suscetibilidade de adolescentes e jovens a riscos de saúde devido ao isolamento e à falta de acesso aos serviços de saúde^(9)^. Durante a pandemia de COVID-19, adolescentes e jovens sobreviventes do câncer em áreas rurais enfrentam disparidades no tratamento oncológico e dificuldades em acessar cuidados e apoio social, afetando sua saúde mental^(29)^. Estudos indicam que esses adolescentes e jovens que vivem em áreas rurais necessitam de apoio na organização de seus deslocamentos e casas de apoio podem ser uma alternativa para facilitar o tratamento oncológico em centros urbanos^(11,28)^.

Nesse contexto, a infraestrutura e os recursos humanos para cuidados de saúde podem variar, substancialmente, entre comunidades rurais e urbanas, com procedimentos de diagnóstico e tratamento frequentemente centralizados nas áreas urbanas^(29)^. O acesso a serviços de saúde é desafiador tanto em centros especializados quanto na atenção primária à saúde. Para otimizar o tratamento do câncer em adolescentes e jovens é importante a colaboração entre os diferentes níveis de saúde, especialmente considerando que o acesso a assistentes sociais e profissionais de saúde mental é limitado nas áreas rurais^(28)^.

A partir do exposto, ficam evidentes as diferentes situações de vulnerabilidade, individual, social e programática diante do tratamento oncológico vivenciado por adolescentes e jovens do contexto rural. Essas diferenças são influenciadas por fatores como acesso limitado à informação sobre saúde, o que pode levar a uma menor conscientização sobre a doença e atrasos no diagnóstico^(20)^. Além disso, o estigma, condições socioeconômicas desfavoráveis, isolamento geográfico e dificuldades de transporte e acesso a recursos educacionais agravam essas situações, dificultando a busca por cuidados de saúde e o compartilhamento de informações sobre a doença^(19,25)^.

Nesse sentido, Ayres define vulnerabilidade como uma característica de grupos suscetíveis a prejuízos, resultante da interação entre indivíduos, como adolescentes e jovens, e seu meio externo^(9)^. A maior vulnerabilidade está associada ao aumento da morbimortalidade e à diminuição da qualidade de vida, sendo mais pronunciada durante transições e grandes mudanças na vida^(27)^. O conceito de vulnerabilidade relaciona-se a riscos e desamparo, decorrentes da exposição a estresses e dificuldades em gerenciar esses riscos, envolvendo fatores externos e internos que afetam a capacidade de lidar com situações adversas^(9)^.

A vulnerabilidade individual, social e programática de pessoas com doenças crônicas é uma questão complexa e crítica de saúde pública. Ayres^(9)^ destaca que a falta de informação sobre a doença aumenta a suscetibilidade ao isolamento e desamparo, afetando não apenas a saúde física, mas também a autoestima, identidade e perspectivas de futuro dos indivíduos.

Adolescentes e jovens do contexto rural precisam deslocar-se para centros urbanos para receber tratamento adequado, enfrentam dificuldades na compreensão de seus diagnósticos e limitações nos serviços locais^(4,29)^. No entanto, destaca-se a falta de políticas públicas e programas de saúde específicos para essa população, como unidades de saúde especializadas e equipes multidisciplinares.

Para enfrentar as situações de vulnerabilidade, é necessário investir em educação promovendo a conscientização sobre o câncer no meio rural, por meio de campanhas de prevenção e informação, visando reduzir o estigma social e promover a detecção precoce da doença^(27)^. Ayres^(9)^ sugere a implementação de políticas públicas que visem à redução das desigualdades sociais e ao fortalecimento da rede de suporte social.

A vulnerabilidade é um tema amplamente discutido em diversos contextos, haja vista a infinidade de usos e referências a essa palavra, no senso comum e nos meios políticos e científicos. Portanto, compreender a vulnerabilidade vai além de um conceito, é um esforço para entender a complexidade da experiência de adolescentes e jovens do contexto rural em tratamento oncológico. A vivência do tratamento oncológico pode se intensificar se eles não conseguirem interpretar seu contexto e experiência com o câncer e agir sobre essa situação criticamente. Em suma, as situações de vulnerabilidade enfrentadas por adolescentes e jovens do contexto rural em tratamento oncológico devem ser compreendidas de maneira integrada, considerando não apenas os aspectos individuais, mas também os coletivos, contextuais, culturais, econômicos e políticos. A valorização desses múltiplos fatores é essencial para desenvolver intervenções eficazes que promovam a saúde e o bem-estar dessas populações vulneráveis.

Assinala-se como limitação do estudo o fato de ter sido desenvolvido em apenas um cenário, o hospitalar, e em apenas uma região geográfica do Brasil. As implicações para a prática em saúde reforçam ser importante que os enfermeiros realizem uma avaliação abrangente da situação individual de cada adolescente ou jovem rural em tratamento oncológico, considerando não apenas sua condição de saúde, mas também fatores sociais, econômicos e geográficos. Este estudo não pretende esgotar este assunto, mas colaborar no aprofundamento e estimular novas pesquisas e provocar reflexões sobre a temática.

## CONCLUSÃO

Os adolescentes e jovens do meio rural em tratamento oncológico enfrentam situações de vulnerabilidade relacionadas à doença, limitações de acesso a cuidados de saúde adequados, dificuldades sociais e emocionais. O diagnóstico de câncer é o primeiro impacto emocional que os deixa suscetíveis à vulnerabilidade individual por não esperarem vivenciar, por um longo período, esse processo de adoecimento na adolescência e juventude. A perda do contato com os amigos, o que faz com que se distanciem, movimenta sentimentos negativos e isolamento. A necessidade de abandonar o trabalho limitou a interação social, privando-os de oportunidades profissionais importantes para seu desenvolvimento pessoal.

Os efeitos colaterais da quimioterapia e as restrições alimentares também representam um impacto significativo. O acesso e o deslocamento para realizar o tratamento foram traduzidos como um desafio para os adolescentes e jovens rurais. Eles também enfrentam reações negativas das pessoas, em seus espaços sociais, fomentando a vulnerabilidade individual e social.

Os resultados do estudo sugerem a necessidade de criar espaços de escuta e acolhimento nos serviços de oncologia, permitindo que os adolescentes e jovens compartilhem suas experiências e necessidades. Isso pode melhorar a qualidade do atendimento e a adesão ao tratamento. O estudo preenche uma lacuna na literatura sobre a saúde de adolescentes e jovens em áreas rurais, contribuindo para um entendimento mais profundo das suas necessidades e desafios. Os achados podem servir como base para novos estudos que explorem outras dimensões da saúde e bem-estar dessa população. Os resultados podem incentivar iniciativas sociais e comunitárias, como grupos de apoio e programas de integração social.
